# The design of the Dutch EASYcare study: a randomised controlled trial on the effectiveness of a problem-based community intervention model for frail elderly people [NCT00105378]

**DOI:** 10.1186/1472-6963-5-65

**Published:** 2005-10-05

**Authors:** René JF Melis, Monique IJ van Eijken, George F Borm, Michel Wensing, Eddy Adang, Eloy H van de Lisdonk, Theo van Achterberg, Marcel GM Olde Rikkert

**Affiliations:** 1 Department of Geriatric Medicine, Radboud University Nijmegen Medical Centre, internal postal code 318, PO box 9101, 6500 HB Nijmegen, The Netherlands; 2 Centre for Quality of Care Research, Radboud University Nijmegen Medical Centre, internal postal code 229, PO box 9101, 6500 HB Nijmegen, The Netherlands; 3 Department of Epidemiology and Biostatistics, Radboud University Nijmegen Medical Centre, internal postal code 252, PO box 9101, 6500 HB Nijmegen, The Netherlands; 4 Department of Medical Technology Assessment, Radboud University Nijmegen Medical Centre, internal postal code 253, PO box 9101, 6500 HB Nijmegen, The Netherlands; 5 Department of General Practice, Radboud University Nijmegen Medical Centre, internal postal code 229, PO box 9101, 6500 HB Nijmegen, The Netherlands

## Abstract

**Background:**

Because of their complex clinical presentations and needs frail elderly people require another approach than people who age without many complications. Several inpatient geriatric health services have proven effectiveness in frail persons. However, the wish to live independently and policies that promote independent living as an answer to population aging call for community intervention models for frail elderly people. Maybe models such as preventive home visits, comprehensive geriatric assessment, and intermediate care qualify, but their efficacy is controversial, especially in frail elderly persons living in the community. With the Dutch EASYcare Study Geriatric Intervention Programme (DGIP) we developed a model to study effectiveness of problem based community intervention models in frail elderly people.

**Methods/Design:**

DGIP is a community intervention model for frail elderly persons where the GP refers elderly patients with a problem in cognition, mood, behaviour, mobility, and nutrition. A geriatric specialist nurse applies a guideline-based intervention with a limited number of follow up visits. The intervention starts with the application of the EASYcare instrument for geriatric screening. The EASYcare instrument assesses (instrumental) activities of daily life, cognition, mood, and includes a goal setting item. During the intervention the nurse regularly consults the referring GP and a geriatrician. Effects on functional performance (Groningen Activity Restriction Scale), health related quality of life (MOS-20), and carer burden (Zarit Burden Interview) are studied in an observer blinded randomised controlled trial. 151 participants were randomised over two treatment arms – DGIP and regular care – using pseudo cluster randomisation. We are currently performing the follow up visits. These visits are planned three and six months after inclusion. Process measures and cost measures will be recorded. Intention to treat analyses will focus on post intervention differences between treatment groups.

**Discussion:**

The design of a trial evaluating the effects of a community intervention model for frail elderly people was presented. The problem-based participant selection procedure satisfied; few patients that the GP referred did not meet our eligibility criteria. The use of standard terminology makes detailed insight into the contents of our intervention possible using terminology others can understand well.

## Background

In frail elderly persons chronic conditions and loss of function challenge their autonomy. This harms their well-being, and often leads to institutionalisation and high health care costs.

There is much heterogeneity in the degree to which frailty affects older people. While some have many problems, others age successfully [1]. The introduction of the concept of successful aging voiced a change in our thinking about 'age-related' decline [2]. It marked the awareness that we cannot simply regard functional loss and dependency as consequences of the aging process itself when disease is absent. With this understanding these 'age-related' deficits became amenable to intervention. Of course, intervention should take the heterogeneity of the population into account; because of their complex clinical presentations and needs frail persons require another approach than people who age without many complications [3]. Although special services for frail older people have proven effectiveness in the form of inpatient geriatric health services [4], several societal developments ask for community equivalents. People prefer to stay at home, even with considerable disability [5]. Another drive behind the development of community intervention models comes from policies that promote independent living as an answer to the questions raised by population aging [6]. Possibly, models such as preventive home visits, in home comprehensive geriatric assessment, and intermediate care provide effective health services for frail older people in the community. Unfortunately, both the definition and efficacy of these community intervention models remain subject of a vivid debate [7-10]. The debate stems from the fact that the models gathered under these names show much heterogeneity as well as considerable overlap [11]. The lack of detailed insight into the content of these care models further complicates comparison [12,13]. One of the major issues is the effectiveness of these models in the expanding group of frail older people.

Despite the diversity, from literature we can distil certain elements that are used in many community intervention models. These are elements such as multidimensional and multidisciplinary working, person centred care, participant selection, and treatment adherence. Empirical evidence is available for some of these elements.

In this paper we will briefly summarise this knowledge on multidimensional assessment and management of elderly people in the community. This information grounds the choices we have made in designing a new community intervention model for frail elderly people living at home. Then, we will present the outlines of our intervention model and the design of the randomised trial in which we are currently evaluating the model. At this moment the recruitment period is already completed, and we are performing the follow up visits. Therefore, in addition to the details of the design, we will highlight some results of the conduct of the recruitment phase of our study.

### Evidence on intermediate care models

Most research has been done on preventive home visits and comprehensive geriatric assessment, less scientific knowledge is available for intermediate care models.

The studies that have been evaluating intermediate care focused mainly on the evaluation of intermediate care alternatives (e.g. rapid response teams, hospital at home, early discharge schemes) in direct comparison with hospital care [11,14]. For most of the models that are not intended as direct alternatives to hospital care (e.g. residential rehabilitation, and community assessment and rehabilitation services) only descriptive data are available [15].

### (Evidence-based) elements of community intervention models

Virtually all community intervention models for older people share a similar multidimensional nature covering a variety of medical, psychological, functional, and social domains. As multidimensional working is a ubiquitous feature of these models, it is in itself not thoroughly studied. There are some discussions on which domains are to be included [8].

Both in preventive home visits and comprehensive geriatric assessment it is suggested that models with a multidisciplinary team are more effective than models with a unidisciplinary approach [8,16]. Effectiveness is also claimed for longer follow up and more home visits, although a recent trial did not confirm this [16,17].

Many models provide person centred care. Some even argue that 'patient-centred, problem-driven, goal-oriented management' is a 'key minimum specification' [16].

Another element that might strengthen the effectiveness of comprehensive geriatric assessment is to secure control over the implementation of the recommendations done in the programme [4]. Models implemented in regular care often do not have complete clinical control over the enforcement of the recommendations following from the programme. In this scenario, it is very important to involve the primary care provider who will be responsible for the implementation of the proposed plan [8].

This is also important because providers' co-operation is a determinant of patient adherence to program recommendations [18]. It is difficult to change physicians' behaviour and this urges the use of high intensity programmes. Furthermore, programme effectiveness might benefit from stronger emphasis on direct recommendations to participants, and should not rely too much on the uptake of recommendations by the primary care provider [8].

Participant selection is a feature of community intervention models for elderly people that received much attention in literature. This discussion focuses on two matters: participant selection on the basis of age and on the basis of participants' needs. Age as a selection criterion is not discussed much, but causes controversy. Some authors state that home visits are more effective in persons aged 75 and over, compared to younger individuals [19]. One meta-analysis did not find an age effect, and another meta-analysis concluded most benefits are to be expected in the youngest old [13,20]. Frailty has received much more attention than age with respect to targeting these health services models to those who will benefit most. Most authors agree that too healthy elderly persons should be excluded, because both preventive home visits and comprehensive geriatric assessment are ineffective in these sprightly people [13,21]. There is more dispute about the effectiveness of these models in frail older persons. While some exclude the frailest participants, because in these persons there are too few possibilities for reversibility, other authors stress the importance of including the frailest [8,13,21,22]. Combining the evidence on the relevance of both age and frailty for participants selection Stuck concludes that health risk appraisal with individual reinforcement is beneficial to healthy persons aged 60 to 75, preventive home visits should focus on independent people aged 75 and over, and that other types of (institutional) services are needed for the frailest [23].

Unfortunately, considered this is true, this conclusion still disregards the population of frail elderly persons living in their own home.

### Considerations on designing the Dutch EASYcare study

We wonder whether the effectiveness of community intervention models for frail elderly people can be enhanced using an alternative way of participant selection. In addition to selecting participants on the basis of age and frailty criteria, we ask the general practitioner (GP) to initiate the intervention when a problem requiring action emerges. This problem-based approach may enhance effectiveness because of better timing of the intervention. Others have shown this type of targeting can be effective, albeit in a non-randomised design [24]. General practitioner's and participant's compliance may also benefit, because both have discussed and agreed on the involvement of another health provider. The general practitioner is directly involved in the intervention model which realises more control over the clinical management. Direct involvement of the GP also provides feedback possibilities to better tailor the intervention and it safeguards continuity of care. We presume this continuity prevents the occurrence of negative effects that could result from discontinuation of the intervention. Hypothetically, the result is that the intensive involvement of health workers than other the general practitioner and regular home care is needed only temporarily.

If an informal carer was involved, we actively engaged this person in our intervention. We believe this involvement is a precondition for an effective community intervention model focussing on frail elderly people. However, to our knowledge, this caregiver involvement has not received much attention in the empirical studies of community intervention models.

### Objectives

The objective of our study is to determine the effects of the Dutch EASYcare Study Geriatric Intervention Programme (DGIP) compared to regular medical care in improving health related quality of life in independently living elderly persons and in improving caregiver burden. Moreover, we want to determine the costs of the Dutch EASYcare Study Geriatric Intervention Programme.

## Methods/Design

### Study design and setting

The study is an observer blinded randomised controlled trial. Pseudo cluster randomisation was used to randomly allocate the participants to the DGIP or to a regular care group. Pseudo cluster randomisation is a randomisation method that aims to prevent both the occurrence of selection bias and contamination in a single design. We will discuss it in more detail below. The Ethical committee of the Radboud University Nijmegen Medical Centre approved of the study.

### Study population

54 general practitioners from 36 GP practices in and around Nijmegen, the Netherlands, were willing to recruit subjects. We started with 38 GPs, but increased this number during the recruitment period because of disappointing inclusion rates. During the inclusion period of 21 months 155 eligible participants were randomised. We decided not to include in follow up and analysis those participants who experienced severe intercurrent disease necessitating hospital admittance, were admitted to a nursing home, died, or withdrew informed consent within one week after randomisation. The possibility of the study to have effect within one week after randomisation was judged as negligible, because it took about a week before nurses started the intervention, and the follow up visits were judged to be too strenuous for these seriously ill patients. Therefore 151 participants were included in follow up and analysis; 85 were included in the group that received the intervention model, and 66 were included in the regular care group.

### Eligibility criteria

Subjects had to be eligible for participation in our intervention model (table 1). All participants had to be living in their own home or in a home for the aged and had to be 70 years or older.

**Table 1:** Eligibility criteria for Dutch EASYcare Study

**Inclusion criteria**

70 years of age and over
The patient lives independently or in a home for the aged
The patient has a health problem that was recently presented to the GP by the patient or informal caregiver
The request for help is related to the following problem fields: cognitive disorders, behavioural and psychological symptoms of dementia, mood disorders, mobility disorders and falling, or malnutrition
The patient/informal caregiver and GP have determined a goal they want to achieve
Fulfil one or more of these criteria: MMSE (Mini Mental State Examination) equal to or less than 26, GARS (Groningen Activity Restriction Scale) equal to or greater than 25 or MOS-20/subscale mental health equal to or less than 75

**Exclusion criteria**

The problem or request for help has an acute nature, urging for action (medical or otherwise) within less than one week
The problem or request for help is merely a medical diagnostic issue, urging for action only physicians (GP or specialist) can offer
MMSE < 20 or proven moderate to severe dementia (Clinical Dementia Rating scale [CDR] > 1, 0) and no informal caregiver (no informal caregiver is defined as: no informal caregiver who meets the patient for at least once a week on average)
The patient receives other forms of intermediate care or health care from a social worker or community-based geriatrician
The patient is already on the waiting list for a nursing home because of the problem the patient is presented with in our study
Life expectancy < 6 months because of terminal illness

When we started recruiting participants we applied an age criterion of 75 years or older. Unfortunately, seven months after the start of the recruitment the inclusion rates fell short of expectations. We decided we were able to broaden the age criterion, because the combination of frailty criteria and a problem driven approach safeguarded selection of eligible participants.

We restricted participant inclusion to those who scored below maximum (indicating good performance) on at least one of the following instruments: Mini Mental State Examination (MMSE), MOS-20 subscale mental health, or Groningen Activity Restriction Scale (GARS) [25-27]. For the MMSE the cut off was equal to or less than 26 out of 30, for MOS-20 mental health equal to or less than 75 out of 100, and for GARS the cut off was equal to or greater than 25. The GARS score ranges 18 to 54, where 18 indicates best functional performance.

We excluded participants with an MMSE of less than 20 or a proved moderate to severe dementia (Clinical Dementia Rating scale [CDR] > 1, 0) and no informal caregiver, because we expected serious problems in the acquisition of research data in these persons.

Persons already receiving forms of intermediate care or health care from a social worker or community-based geriatrician were also excluded, because this made it difficult to establish which effect was measured. Receiving home care, however, was not an exclusion criterion.

Persons already on the waiting list for a nursing home, or who had a life expectancy of less than six months, because of terminal illness, were excluded as well.

As a result of a mistake, in one case the age criterion was violated. However, the intervention team agreed that this younger case (age of this participant was 69 years) fitted well into the model. As exclusion was judged to be in disagreement with the ethical treatment of participant data, this participant was kept in follow up and analysis.

### Treatment arms and randomisation

Participants were randomly allocated over two treatments arms: DGIP and regular care. No restrictions were imposed on the care participants were allowed to receive in the regular care group.

Given the nature of our intervention we considered the use of two different allocation procedures available in literature: cluster randomisation or individual randomisation [28]. The use of a cluster randomised design may have had an advantage over the use of an individual randomised design, because of the possible occurrence of contamination in our trial when individual randomisation was applied [29]. On the other hand a cluster randomised design had several disadvantages. The GP would have known the allocation outcome for his cluster after the first patient in a fully cluster randomised design. This might have caused selection bias resulting in incomparability of treatment arms [30,31]. At the same time we presumed it likely that the recruitment of subjects in the control clusters would progress slowly. Why should a GP bother to refer a patient to a study, when the GP knows already that the patient will enter the control group? There is also evidence for differential recruitment rates in cluster randomisation [32].

We therefore choose to use an innovative two-step pseudo cluster randomisation procedure [28,33]. First the GPs were randomised into two groups; group I and group C. The results of this randomisation were not revealed. Then within each of these groups randomisation at the patient level was carried out. This randomisation was stratified by GP and performed in such a way that in group I the majority (approximately 80%) of the participants received the intervention treatment, while the others received standard treatment. In group C the dysbalance was reversed: the majority received standard treatment and the others got the intervention treatment.

This approach had important advantages. The GP did not know in advance which treatment a patient was going to get, so this reduced the chance of selection bias. It also prevented the occurrence of negative recruitment effects that might have resulted from being randomised to a control cluster. Had the GPs known in advance the group they were assigned to (I or C), the predictability of an individual randomisation decision had been larger than in an individually randomised trial. However, the randomisation of GPs occurred blinded. In such a situation, the GP can only gain knowledge on the randomisation proportion through the recruitment of participants. As the number of enrolled patients per practice was expected to be no more than 10, the chances to correctly guess the odds for each individual treatment are limited.

We expect the contamination due to the intervention treatment to be negligible in group C, because there are only a limited number of participants in this group on the experimental treatment. As the majority of the patients is on intervention treatment, the contamination may be a problem in patients in group I who are on standard treatment, but then it probably affects only a small portion of the patients.

A randomisation procedure with adaptive weights (minimisation) was used to ensure a balanced distribution of high versus low percentage of elderly per GP-practice and of the availability of a nurse practitioner in GP practice in the two groups I and C [34]. The patients were randomised with adaptive weights to get evenly distributed numbers of sex, and presented health problem. A person not related to the study conduct performed the randomisation.

### Intervention model: DGIP

GPs referred independently living older patients to our model when there was a problem in cognition, nutrition, behaviour, mood, or mobility. The problem had to urge for nursing assessment, co-ordination of care, or therapeutic monitoring and case management. Requests were rejected if they had an acute nature or if they were purely medical diagnostic requests.

A suitable case is for example a widow living on her own in a flat on the second floor with no elevator. The GP has doubts about her cognitive abilities and she has depressive symptoms as well. This seems to affect her daily functioning, although to what extent is unclear. She has only a daughter to look after her.

After negotiating a preliminary goal with the patient, the referring GP contacted the geriatrician involved in the study. Within two weeks a geriatric specialist nurse visited the patient at home. The instrument EASYcare was applied during this first visit [35]. EASYcare is an instrument for geriatric assessment that consists of items about (instrumental) activities of daily life, cognition, mood, and ends with a goal setting item. The goal initially negotiated by patient and GP was further elaborated in an operational objective. If an informal carer was present, the nurse provided this person a carer burden assessment and the results were implemented in the care plan.

During maximum three months up to five follow up visits for additional geriatric evaluation and management were planned. The nurse, geriatrician, and general practitioner frequently discussed the necessary nursing interventions, the effect of the interventions, the level of care that was needed, and the possibilities for reversibility. If necessary the nurse consulted and advised other involved health care workers, such as home care or physical therapist.

We had two nurses and two geriatricians available for the execution of our intervention. We developed guidelines based on best nursing practice for each health problem to structure activities, because literature has pointed at the possibility that the effects of home visiting programmes are related to the home visitor's performance in conducting the visits [36]. Therefore, we structured the intervention in order to diminish this effect, without harming the flexibility of the model. Our guidelines divided the nursing process into four phases: nursing diagnosis, definition of expected outcomes, nursing interventions and assessment of outcomes. Secondly, the guidelines used standardised NANDA (North American Nursing Diagnosis Association), NOC (Nursing outcomes classification) and NIC (Nursing interventions classification) terminology for nursing diagnosis, nursing outcomes and nursing interventions respectively [37-39].

We piloted our intervention model in a feasibility study [40]. With some minor changes, this model was judged to be applicable in the current study.

### Data collection and outcome measures

Within one week after referral a researcher (RM, ME) interviewed patients at home to obtain written informed consent and to collect baseline demographic characteristics and data on general health conditions. If the participant was not able to give informed consent we asked a proxy to do so. The participants always gave verbal assent and did not reject the measurements. Before the interview the participant received a written confirmation of the appointment and a questionnaire. We asked the participant to fill out the questionnaire before the appointment. If the participant was unable to fill out the questionnaire independently, we allowed help from another person. In some cases the interviewer filled out the questionnaire during the interview. We recorded the amount of help the participant received in filling out the questionnaire.

The participants provided data on the following measures: age, gender, type of residence, and the use of home care. Also, data were collected on functional abilities, cognitive condition, mobility, health-related quality of life, and loneliness.

If an informal carer was available we collected data on informal carer characteristics using a questionnaire. We collected data on type and amount of care provided, time spent on caring, and carer burden.

These measurements are repeated three and six months after inclusion. The same researcher that performed the baseline visit carries out these interviews. This researcher is not involved in the intervention nor does the researcher know the allocation decision. After each follow up visit the researcher indicates whether blinding remained intact or not.

Primary outcome measures relating to participant characteristics are functional performance in (independent) activities of daily living measured using Groningen Activity Restriction Scale and mental health using subscale mental health MOS-20. Primary outcome measure in informal carers is carer burden using the Zarit Burden Interview (ZBI) [41]. An overview of secondary outcomes and a complete list of all measurements are provided in table 2.

**Table 2:** Outcome measures

**Variable**				**Instrument**			
**Background variable**							

**Secondary outcome**							

**Primary outcome**				**Measured at**	**T**_0_	**T**_1_	**T**_2_

Functional performance (ADL/IADL)				GARS-3 [27]			
• Mobility				Timed up and go test [44]			
Health Related quality of life				MOS-20 [26]			
Mood				Subscale mental health MOS-20			
Well-being				Cantril self-anchoring ladder [45]			
				Dementia Quality of Life questionnaire [46]			
				question general life satisfaction			
Cognition				MMSE [25]			
Social functioning				Loneliness scale de Jong-Gierveld [47]			
Mortality							
Housing conditions/sort of residence				Own questionnaire			
Subjective treatment effects (participant, informal carer)				Patient Enablement Instrument [48]			
Burden informal carer				Zarit Burden Interview [41]			
				Questions taken from 'Zorgkompas Mantelzorger' [49]			
Time spend on care (informal carer)				Own questionnaire			
Age (participant, informal carer)				Own questionnaire			
Sex (participant, informal carer)				Own questionnaire			
Socio-economic status				Own questionnaire, classify using ISEI-92 [50]			
• (Former) occupation				Own questionnaire, classify using SBC-92 [50]			
Nativity				Own questionnaire			
Co-morbidity				Cumulative Illness Rating Scale-Geriatrics (CIRS-G) [51] from medical history in GP Information System			
Use of home care				Own questionnaire			

T_0 _is baseline measurement

T_1 _is first follow up measurement, after 3 months

T_2 _is second follow up measurement, after 6 months

### Process evaluation

We collect data on the following set of process variables: the content of the intervention programme, the adherence of participants and informal carers in the intervention group to advices given during an intervention, experiences of participants and informal carers with the intervention model, and data on GP care and care of other involved professionals in both treatment arms.

We collect data on the content of the intervention process, because this may help to identify which programme characteristics are most beneficial. An abstract form is used to extract this information from the nursing records after completion of all individual interventions. We extract information on treatment goals, nursing diagnoses (NANDA) [37], nursing interventions (NIC) [38], nursing outcomes (NOC) [39], and the employed diagnostic instruments.

Compliance of participants and informal carers is an important determinant of carrying out a successful intervention. When an individual intervention is finished the nurse that executed the intervention indicates in an MS Access^® ^form which of a number of pre-specified advices were given. Another nurse calls the participant or informal carer one month later to check compliance on these advices.

We score subjective treatment effects in treatment group using a questionnaire that participants and informal carers filled out after the first follow up visit.

Data on GP care will be collected in both treatment arms from the information that is routinely available from the General Practice's Information System (Huisartsen Informatie Systeem). We collect the following data: medical history using ICPC-2 (International Classification of Primary Care) [42], number and content of contacts during six months of follow up using ICPC-2, number and nature of referrals, and medication using ATC classification (Anatomical Therapeutic Chemical drug classification) [43]. Data on the use of home care are collected in the participant questionnaire. The data on GP care will be collected at the end of the follow up period. These data are collected in order to be able to clarify the observed intervention effect and to establish costs.

### Costs

To be able to calculate costs, data will be collected on the following cost variables. Nurses will register the time spent on the intervention using the MS Outlook^® ^agenda. They will register the number of visits per participant. They also register the time spent on consultation, phone calls, travelling, and administration.

Data on the workload of the GP and the geriatrician will be extrapolated from the workload of the nurses. The data we collect on the care provided were already described in the paragraph 'process evaluation'. Finally, we will derive salary costs, administrative costs, and costs for materials.

### Sample size considerations

A change in the primary outcome measure of functional performance (GARS-3) of 4.5 points on a scale ranging from 18 (complete independence) to 54 (complete dependence) can be found with a power (1-β) of 0.80 and α (two sided) of 0.05 in comparing two groups of 77 subjects, when pseudo cluster randomisation is applied. We use a standard deviation of 8.5, which we calculated from a pilot study. This standard deviation is well in the range of the measures of spread other studies have found [27]. A mean increase of 4.5 points is chosen as clinically relevant, because a 4.5 point increase of the overall score indicates an improvement of 25% of all items by one functional class (each item's score is classified as follows: completely dependent 3 point, partly dependent 2 points and completely independent 1 point). Cluster size is estimated to be approximately 10 participants per GP. The exact calculations and considerations are extensively described in Teerenstra et al [33].

### Statistical analysis

Descriptives will be used to assess comparability of both intervention and control group for background and confounding variables. Our primary analysis will focus on the treatment arms' differences in the primary outcome measures' changes from baseline (GARS, MOS-20 subscale mental health, and Zarit Burden Interview) at three months of follow up (T_1_). This will be done in intention-treat-analysis. We will use mixed linear model analysis (Proc Mixed in SAS^® ^8) to quantify these differences. We will account for clustering at the level of the GP through the addition of a random intercept for GP to the three models. The baseline measurements of GARS, MOS-20 subscale mental health, and Zarit Burden Interview will be added to the respective models as a covariate. The factors we stratified for in the randomisation (GP-characteristics, sex of participant, and participant's presented health problem) will also be added to the models as covariates. No further corrections will be made. A conditional analysis of the treatment arms' differences in changes from baseline at six months (T_2_) will be performed if there is a significant effect at T_1_. Apart from replacing the scores at three months with those at six months the same three models will be used.

The secondary analyses will be performed on the treatment arms' differences in time trend of the primary outcome measures GARS, MOS-20 subscale mental health, and Zarit Burden Interview during follow up. Secondary analysis will further focus on the differences between treatment arms of the secondary outcome measures at three and six months of follow up. Kaplan-Meier estimates and hazard ratios will be used to quantify the intervention's effect on living conditions and mortality. Subgroup analyses will be performed for the following subgroups: living in one's own home versus living in a home for the aged, and higher versus lower levels of cognitive function. All analyses will be performed in SAS^® ^8.

## Discussion

In this paper we presented the design of a randomised controlled trial that evaluates the effects of a community intervention model for frail elderly people living on their own. The design of this study has shown to be very challenging.

Although the recruitment of the participants took much effort, we have included a number of subjects that should be large enough to provide reliable answers to our research questions.

Our participants were selected using a problem-based approach in which the GPs decided in co-operation with the geriatrician which patients were suitable for this intervention model. This participant selection procedure satisfied; only a minor number of the referred patients did not meet our eligibility criteria based on frailty and age. Probably, piloting our intervention model was important to achieve this.

As discussed earlier, there is a lack of insight into the content of most community intervention models studied. We decided to use standard terminology such as ICPC, NANDA, NIC, NOC and ATC codes to provide insight into our intervention when used in practice. This makes detailed insight possible using terminology others can understand well.

The selection of the best randomisation method was a final major issue we had to deal with and that took much of our time. We think this randomisation procedure satisfies. Nevertheless, we will closely monitor and report in future papers how the randomisation procedure performs in practice.

Dissemination of the results of this study is planned for 2006.

## Competing interests

The author(s) declare that they have no competing interests.

## Authors' contributions

MO and RM were responsible for the research question. RM, ME, TA, and MO designed the study. RM and ME carried out the data acquisition. RM wrote the first draft of this manuscript and was responsible for the revisions. ME, TA and MO contributed to drafting of the manuscript. GB gave advices on the statistical analysis, EA on the economic analysis. MW, EL, GB and EA commented on the design and the manuscript. All authors read and approved the final manuscript.

**Figure 1:**
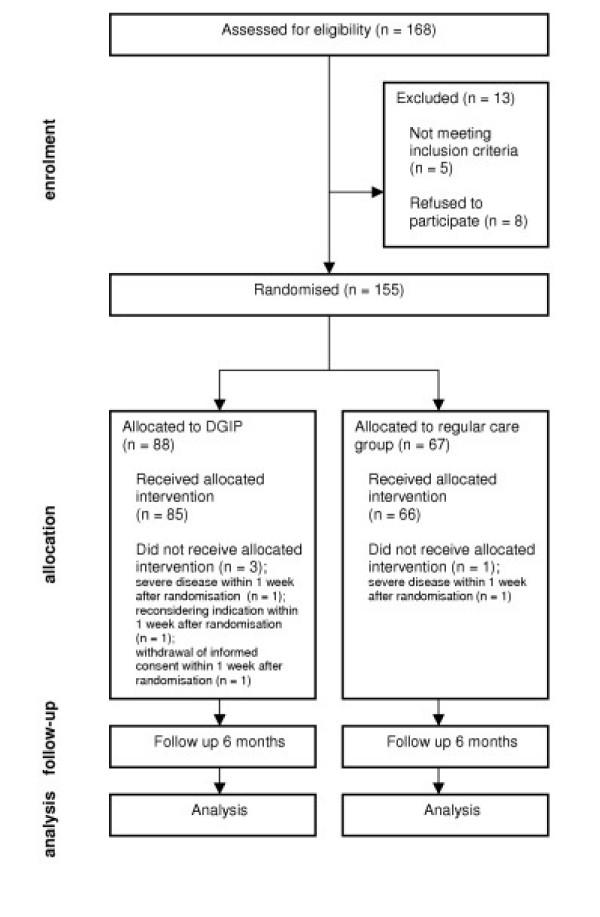
flow chart Dutch EASYcare Study. This flow chart summarises the progress through the phases of the Dutch EASYcare Study until the allocation of participants to each treatment arm.

## Pre-publication history

The pre-publication history for this paper can be accessed here:


